# The Role of Emotional Intelligence on Psychological Adjustment and Peer Victimization in a Sample of Spanish Adolescents

**DOI:** 10.3389/fpsyg.2020.600972

**Published:** 2020-12-18

**Authors:** Elizabeth Cañas, Jesús F. Estévez, Estefanía Estévez, David Aparisi

**Affiliations:** ^1^Department of Health Psychology, Miguel Hernández University of Elche, Elche, Spain; ^2^Department of Developmental Psychology and Didactics, University of Alicante, Alicante, Spain

**Keywords:** victimization, self-concept, life satisfaction, emotional intelligence, moderating effect

## Abstract

In the last decades, interest in the study of the negative consequences of bullying for the victims has increased. Victims are often known to show emotional adjustment issues, such as negative self-concept and low life satisfaction. Moreover, some studies have observed important associations between self-concept and life satisfaction, in which a positive self-concept is related to high levels of life satisfaction. Other studies have pointed out the importance of emotional intelligence (EI), as a regulatory and protective factor against the negative impact of victimization on adjustment in adolescents. The main objective of this work was to analyze the mediating effect of self-concept on life satisfaction and the moderated mediation effect of EI on self-concept and life satisfaction in the context of peer victimization. The participants in the study were 1,318 Spanish students of both sexes and aged between 11 and 18 (*M* = 13.8, SD = 1.32) years, from four compulsory secondary education centers. The results indicated that, on the one hand, self-concept mediated the relationship between victimization and life satisfaction. On the other hand, EI was not only positively associated with self-concept, but it also significantly moderated the negative influence of victimization on self-concept. EI may also indirectly moderate the relationship between victimization and life satisfaction through the self-concept. These data show the importance of EI as a possible protective and moderating factor of the negative effect of bullying on emotional adjustment, which is interesting for the design of future prevention and intervention programs in school contexts.

## Introduction

In the last decades, the school context is facing an important social problem that is related to violent behavior among adolescents, known as peer victimization (Rey et al., 2019). Peer victimization involves receiving any acts of aggression from similar-age peers (Wu et al., 2015). This victimization can occur directly through verbal or physical aggressions, or indirectly, using more subtle forms of attack through actions that include social exclusion or peer rejection, spreading rumors, and threats to withdraw friendship (Mehari and Farrell, 2015; García et al., 2017). Victimization rates in young population require close attention, since according to the data collected by the World Health Organization (WHO) in the Global School-based Student Health Survey (from 2003 to 2017) in collaboration with UNESCO (2018), the proportion of students who report being bullied is 33% in those aged 13, 32.3% aged in those aged 14 and 30.4% in those aged 15 years. Besides, they are more likely to experience negative consequences associated to this problem. Research conducted until now shows that victimization negatively affects the emotional adjustment, health, and well-being of the victims (Menesini and Salmivalli, 2017).

### Relationship Between Victimization and Emotional Adjustment

Recent research with adolescent samples has considered negative self-concept as an important emotional consequence associated with peer victimization (Norrington, 2020). Self-concept is defined as the subject’s perceptions of the self in different domains, such as academic, social, family, and physical dimensions (García and Musitu, 1999). The growing number of studies focusing on the indicators of emotional maladjustment in victimization has suggested that most adolescents who are bullied tend to perceive themselves negatively (Malhi et al., 2014; Cañas et al., 2019) and, in consequence, to experience negative self-concept (Cañas et al., 2020). In this vein, some studies have found that adolescents’ assessment of their self-concept is intimately related to their overall assessment of their lives, so a positive self-concept is associated with high levels of life satisfaction (Chui and Wong, 2016; Ortuño-Sierra et al., 2019; Povedano-Diaz et al., 2020).

Particularly, life satisfaction has been considered as an indicator of the overall quality of life (Diener et al., 1999), as well as of well-being (Diener et al., 1999). Like self-concept, well-being has been also negatively associated with victimization in adolescence (Callaghan et al., 2015; Weng et al., 2017; Gini et al., 2018; Cañas et al., 2020). Additionally, some works have indicated that victimization has a negative influence on the victims’ well-being (Låftman and Modin, 2017; Callaghan et al., 2019), so it is not surprising that bullying victims are significantly more likely to experience low life satisfaction. For this reason, some investigations into youth violence have considered victimization as a salient risk factor for poor life satisfaction in adolescence (Méndez-Giménez et al., 2017; Gini et al., 2018).

Thus, according to empirical evidence, self-concept and life satisfaction are significantly related in adolescence (Carrascosa et al., 2018; Estévez et al., 2019a).

### The Mediating Effect of Self-Concept on Victimization and Life Satisfaction

In the context of peer victimization, Norrington (2020) carried out a longitudinal study about the effect of self-concept on psychological distress in victims of bullying, revealing that self-concept may act as a mediator in the relationship between victimization and mental health. Norrington’s study is relevant when considering that mental health is usually measured evaluating life satisfaction (Kardefelt-Winther and Maternowska, 2020). In short, these findings seem to confirm that self-concept during adolescence plays an important role in the perception of adolescents’ life satisfaction. However, in the field of peer victimization, research focused on the relationship between self-concept and life satisfaction in adolescents is still limited.

Considering the previous findings, it is important to emphasize that self-concept and life satisfaction, separately, comprise an emerging area of inquiry related to victimization in youths (Navarro et al., 2015; Gini et al., 2018; Miranda et al., 2019). Besides, the available results help to understand how self-knowledge and ego-building are key elements for adolescent well-being (Povedano-Diaz et al., 2020), which can be indicative of life satisfaction. However, thus far, there is little research on the mechanism linking self-concept to life satisfaction in the context of peer victimization in adolescents. The moderating effect of emotional intelligence (EI).

The study of the role of emotion regulation in peer victimization has increased in recent years, because it is considered a protective factor against the negative impact of bullying during adolescence (Zych et al., 2017; Quintana-Orts et al., 2019). One of the approaches with a stronger theoretical and empirical basis concerning emotion regulation is the concept of EI, which refers to the ability to perceive, assimilate, understand, and regulate emotions (Salovey and Mayer, 1990). EI also includes motivating oneself and recognizing and managing emotions, both regarding oneself and others (Goleman, 1995). The model proposed by Salovey and Mayer (1990) highlights three components of general EI: (1) attention – the ability to perceive one’s own and others’ emotions; (2) clarity – the ability to understand emotional information, how emotions combine and change over time, and to appraise emotional meanings; and (3) regulation – remaining open to feelings and monitoring and regulating emotions to promote understanding and personal growth.

Regarding the protective mechanism of EI, research has shown that youths with high levels of EI are more likely to cope with negative experiences than their peers with lower levels of EI (Estévez et al., 2019c). This effect has been attributed to better affective processes, which allow maintaining positive mental states, promoting emotional adaptation, and regulating negative moods when faced with threatening situations (Gómez-Baya et al., 2017; Martínez-Monteagudo et al., 2019). Consequently, at high levels, EI may act as a potent construct for the promotion of emotional adjustment and well-being (De la Barrera et al., 2019). In this vein, some studies point out that adolescents with high levels of EI tend to maintain a positive self-concept (Martínez-Monteagudo et al., 2019; Suriá-Martínez et al., 2019) and to have high life satisfaction (Sun et al., 2014; Lázaro-Visa et al., 2019; Ramos-Díaz et al., 2019). Although it is clear that EI has a significant influence on self-concept and life-satisfaction, there has thus far been limited research examining the moderating mechanism of EI in these variables in samples of adolescents.

Concerning victimization, some works have suggested that EI may be an important protective factor against the detrimental effects of victimization, because it provides positive emotional management tools and resources to cope with this stressful situation (Domínguez-García and Fernández-Berrocal, 2018; Extremera et al., 2018; Estévez et al., 2019c; Quintana-Orts et al., 2019). However, victims of bullying generally tend to show low levels of EI (Estévez et al., 2019a; Cañas et al., 2020), and to experience problems of emotional adjustment (Lomas et al., 2012). Perhaps the low EI levels reported by victims are the cause of their manifestations of the negative consequences of victimization. Additionally, it should be noted that not all victimized youth develop the same negative consequences or with the same degree of intensity (García et al., 2020). Recent studies have suggested that differences in the consequences or their intensity could be attributed to the degree to which the person concerned has developed EI (Extremera et al., 2018). Although the available research is focused on cyberbullying, the results underline that EI may not only be a mediator of emotional adjustment but may also be a potential moderator in the relationship between victimization and the associated emotional problems (García et al., 2020). These data support the idea that EI may act as a buffer against the negative impact of victimization (Extremera et al., 2018). Despite the increasing attention given to the relationship between EI and victimization in recent years, the moderating role of EI on individual factors such as self-concept and life satisfaction in the peer victimization context is still not fully understood.

### The Present Study

Based on the reviewed literature, there is evidence suggesting that victimization negatively influences the victims’ emotional adjustment and well-being, such as self-concept (Turner et al., 2017) and life satisfaction (Varela et al., 2017). There is also evidence that self-concept is intimately related to life satisfaction (Chui and Wong, 2016; Ortuño-Sierra et al., 2019; Povedano-Diaz et al., 2020), but, until now, no study has investigated whether self-concept mediates the relationship between victimization and life satisfaction. Similarly, even though the literature pays considerable attention to the relationship between EI and victimization (Zych et al., 2017; Estévez et al., 2019a), there are still deficiencies in the study of EI as a potential moderator of self-concept and life satisfaction in the context of peer victimization. This study proposes moving in this direction to fill the gaps in these issues. Thus, the objectives of the present study were: (1) to examine a mediation model that investigates the effect of self-concept on life satisfaction and victimization; (2) to analyze a moderated mediation model that studies the role of EI on the relationship between victimization and self-concept and life satisfaction. Based on the previously reviewed research on victimization in adolescence, the following hypotheses were established: (1) self-concept would mediate the association between victimization and life satisfaction; (2) EI would moderate the direct and indirect effect of victimization on self-concept and life satisfaction.

## Materials and Methods

### Participants

Analyses of this study are based on data from a representative sample of secondary school students who were recruited through probabilistic sampling, using as primary sampling units the urban geographical areas of the provinces of Alicante, Valencia, Seville, and Teruel, and as secondary units, the public schools in each area. The grades or classrooms were not used as tertiary units, as all the students of the four courses of Compulsory Secondary Education (CSE) in all the schools participated. The socioeconomic level of the areas and schools was average. Approximately, the percentage of the parents of the participating students that had primary education, secondary education, high school studies, or university studies was equitable (25%). Most of the parents performed paid work outside the home: 86.7% of the fathers and 69.5% of the mothers. The final sample was composed of 1318 adolescents (47% boys and 53% girls), aged between 11 and 18 years (*M* = 13.8, SD = 1.32), and enrolled in four CSE schools in the Andalusian, Aragonese, and Valencian communities, in Spain. Students’ distribution by academic grade was balanced: 24.7% were enrolled in 1st grade of CSE, 27.3% in 2nd grade, 23.7% in 3rd grade, and 24.3% in 4th grade.

### Instruments

#### Peer Victimization

To identify victims of bullying, the self-report measure *Peer Victimization Scale* (PVS; Mynard and Joseph, 2000), was used. The scale was translated into Spanish, using the parallel back-translation procedure of Brislin (1986). This 22-item instrument measures three peer victimization dimensions: relational victimization (e.g., “A classmate tried to get me into trouble with my friends”), overt physical Victimization (e.g., “A classmate beat me up”), and overt verbal victimization (e.g., “A classmate called me names”). Rated on a 4-point Likert-type scale (1 = *never*; 4 = *always*), the Cronbach alphas of the three dimensions in the present sample were 0.92, 0.69, and 0.88, respectively, and 0.95 for the global scale.

#### Self-Concept

The global dimension and four dimensions of self-concept were measured using *the Self-Concept Form-5 Scale* (AF5; García and Musitu, 1999). This 24-item scale measures four dimensions of self-concept (6 items per dimension): academic (e.g., “I work a lot in class”), social (e.g., “I have trouble talking to strangers”), family (e.g., “I am very happy at home”), and physical (e.g., “I take care of myself”). The response scale ranges from 0 (*strongly disagree*) to 9 (*strongly agree*). The Cronbach alpha in the present study was 0.89 for the global scale (academic 0.90; social 0.76; family 0.86, and physical 0.79).

#### Satisfaction With Life

Life satisfaction was measured using the *Satisfaction with Life Scale* (Diener et al., 1985) adapted to Spanish by Atienza et al. (2000). This instrument contains 5 items that provide a general index of subjective perceived well-being (e.g., “I’m not happy with my life”). The items are rated on a four-point Likert-type scale ranging from 1 (*strongly disagree)* to 5 (*strongly agree)*. In this study, the Cronbach alpha was 0.78.

#### Perceived Emotional Intelligence

Emotional intelligence was measured using the *Perceived Emotional Intelligence Scale* (TMMS, Salovey et al., 1995) adapted to Spanish by Domínguez et al. (2010). This scale consists of 22 items with 5 Likert-type response options (*strongly agree* to *strongly disagree*), which provide a measure of EI based on three dimensions: emotional attention (e.g., “I think about my mood constantly”), emotional clarity (e.g., “Often, I am mistaken about my feelings”), and emotion regulation (e.g., “Although I sometimes feel sad, I have an optimistic viewpoint”). The global Cronbach alpha of this study was 0.91, and for the dimensions, it was 0.89, 0.86, and 0.87, respectively.

### Procedure

Data of this research were collected as part of a larger study on psychological adjustment in adolescence in Spain. After receiving authorization for the study from the Ethics Committee of the Miguel Hernández University, research assistants sent a letter with a summary of the research project to the headmasters of the selected schools as a first step. Subsequently, initial telephone contact with them was established, followed by a meeting with all the teaching staff in which the objectives of the study and the procedure to be followed for data collection were reported. After the staff had agreed to participate, an explanatory letter was sent to the parents, requesting them to indicate in writing if they did not wish their child to participate in (1% of parents used this option). The administration of the instruments was carried out by a group of trained and expert researchers in each region. Before data collection, students also attended a short briefing in which they provided written consent (none of the adolescents refused to participate). On the dates scheduled with the teaching staff, students were approached in their school classrooms to fill out the questionnaires voluntarily during a regular class period. The order of administration of the instruments was counterbalanced in each classroom and school. The instructions were read aloud, emphasizing the importance of answering all questions and the anonymity of the answers. During the administration of the tests, the researchers were present to resolve doubts and ensure an unbiased process. The surveys that were suspicious in terms of the response patterns were not coded in the database (these surveys represented 1% of the total original samples). Finally, a class of approximately 55 min were required for data collection.

### Statistical Analyses

Firstly, we examined whether the data followed a normal distribution. As Table 1 shows, the skewness and kurtosis of victimization, self-concept, life satisfaction, and EI fell within the acceptable range, with skewness < | 2.0| and kurtosis < | 7.0| (Hancock et al., 2018). Therefore, the variables were used directly in subsequent analyzes. A moderated mediation model was developed through various steps to test the hypotheses. Firstly, descriptive statistics and Pearson correlations among the study variables were calculated (Table 1). Secondly, PROCESS macro for SPSS (Hayes, 2018) was used, applying Model 4 to examine the mediating effect of self-concept between victimization and life satisfaction (Table 2). Thirdly, Model 58 was used to examine the moderating effect of EI on the relationship between victimization and self-concept on one hand, and self-concept and life satisfaction, on the other (Table 3). Sex and age were entered to control their effect on the results. The predictor variables that defined the products were mean-centered to avoid non-essential multicollinearity (Frazier et al., 2004; Fairchild and Mcquillin, 2010). The bootstrap confidence intervals (CIs) helped to determine whether the effects in Model 4 and Model 58 were significant at the α = 0.05 level of significance, based on 5000 random samples. An effect is regarded as significant if the CI does not include zero.

**TABLE 1:** Pearson correlations and descriptive statistics of the variables.

Variables	1	2	3	4
1. Victimization (1–4)	1.00			
2. Self-concept (0–9)	−0.23**	1.00		
3. Life satisfaction (1–5)	−0.34**	0.55**	1.00	
4. Emotional intelligence (1–5)	–0.05	0.38**	0.30**	1.00
Mean	1.61	6.39	3.73	3.32
Standard deviation	0.52	1.25	0.86	0.71
Skewness	1.23	–0.77	–0.47	–0.08
Kurtosis	1.46	0.42	–0.48	0.04

**p < 0.01.

**TABLE 2:** Coefficients for the tested mediation model between victimization and life satisfaction.

Independent and mediation variables	Dependent variable: self-concept β (95% CI)	Dependent variable: life satisfaction β (95% CI)
Victimization	−0.55** (−0.68, −0.43)	−0.39** (−0.46, −0.31)
Self-concept		0.34** (0.31, 0.38)
Age	−0.18** (−0.23, −0.13)	−0.03 (−0.54, 0.02)
Sex	0.01 (−0.11, 0.14)	0.10** (0.02, 0.17)
Constant	9.80** (9.12, 10.48)	2.47** (1.96, 2.98)
	*R*^2^ = 0.09	*R*^2^ = 0.36
	*F*(3,1300) = 44.27, *p* < 0.01	*F*(4,1299) = 181.17, *p* < 0.01

**p < 0.01.

**TABLE 3:** Coefficients for the tested moderated mediation model linking victimization, self-concept, life satisfaction, and emotional intelligence.

Independent and mediation variables	Dependent variable: self-concept β (95% CI)	Dependent variable: life satisfaction β (95% CI)
Victimization (V)	−0.51** (−0.62, −0.39)	−0.39** (−0.47, −0.32)
Emotional intelligence (EI)	0.64** (0.56, 0.73)	0.16** (0.10, 0.22)
EI × V	0.21** (0.6, 0.36)	
Age	−0.18** (−0.23, −0.14)	−0.03* (−0.06, −0.01)
Sex	0.07 (−0.04, 0.18)	0.11** (0.04, 0.19)
Self-concept (SC)		0.31** (0.27, 0.35)
EI × SC		0.02 (−0.02, 0.06)
Constant	2.52** (1.92, 3.11)	4.15** (3.76, 4.54)
	*R*^2^ = 0.23	*R*^2^ = 0.37
	*F*(4,1313) = 78.12, *p* < 0.01	*F*(6,1297) = 128.49, *p* < 0.01

*p < 0.05; **p < 0.01; EI × V, interaction between emotional intelligence and victimization; EI × SC, interaction between emotional intelligence and self-concept.

## Results

### Preliminary Analysis

Table 1 shows means, standard deviations, and Pearson correlations for the studied variables. Victimization was negatively correlated with self-concept and life satisfaction and showed an almost null correlation with EI. The rest of the variables in the study showed positive and significant correlations with each other. The highest coefficient was observed between self-concept and life satisfaction.

### Testing for Mediation Effect

**FIGURE 1:**
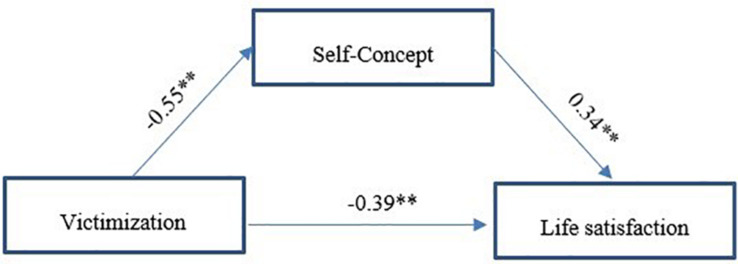
Proposed mediation model linking victimization, self-concept, and life satisfaction. ^∗∗^*p* < 0.01.

A Process Macro Model 4 was built to test the mediation effect, which is illustrated in Figure 1.

Table 2 shows two columns: the first reveals the relationship between the independent variable and the mediator, and the second shows all the coefficients in a simple mediation model. The first column in this table indicated that victimization and self-concept were significantly and negatively related (β = −0.55, *p* < 0.01). However, the percentage of explained self-concept variance was very small (*R*^2^ = 0.09), suggesting the existence of other explanatory variables that had not been introduced in the model. The last column of Table 2 presents the complete mediation model that relates victimization and life satisfaction including self-concept as mediator. This model showed a statistically significant and negative relationship between victimization and life satisfaction (β = −0.39, *p* < 0.01). Besides, the percentage of explained self-concept variance, as the mediator, increased in this model compared to the previous model (*R*^2^ = 0.36), suggesting that self-concept plays an important explanatory role in this model. Self-concept also showed a significant and positive coefficient with life-satisfaction (β = 0.34, *p* < 0.01). The model estimated that the total effect of victimization on life satisfaction was −0.58 [95% CI (−0.66, −0.49)], and showed a significant indirect effect of victimization through self-concept [β = −0.19, 95% CI (−0.24, −0.14)].

In summary, the analysis revealed that self-concept can act as a mediator of the relationship between victimization and life satisfaction, such that victimization negatively affects self-concept, and this, in turn, affects victims’ life satisfaction.

Testing the moderated mediation effect of EI. The results of the EI moderated mediation analysis are presented in Table 3. Victimization again showed a negative relationship with self-concept in the model that used self-concept as a dependent variable (β = −0.51, *p* < 0.01). However, EI had a positive coefficient with self-concept (β = 0.64, *p* < 0.01). Furthermore, the interaction between EI and victimization (EI × V), which indicated whether or not there was a moderating effect, showed a positive and highly significant coefficient, indicating that EI could act in two ways in this relationship between victimization and self-concept. The first way through its positive direct relationship with self-concept (β = 0.64, *p* < 0.01), and the second way by exerting a moderating effect on the negative influence of victimization in self-concept (β = 0.21, *p* < 0.01). In contrast, the last column of Table 3 showed that EI did not have a moderating effect on the relationship between self-concept and victimization (EI × SC, *r* = 0.02, *p* > 0.05), but it did have a direct relationship with life satisfaction (β = 0.16, *p* < 0.01). Thus, these results indicated that EI was positively related both to self-concept and life satisfaction, and that EI moderated the influence of victimization on self-concept. In this sense, although no evidence was found that EI directly moderated the relationship between victimization and life satisfaction, it can indirectly moderate this relationship through self-concept.

**FIGURE 2:**
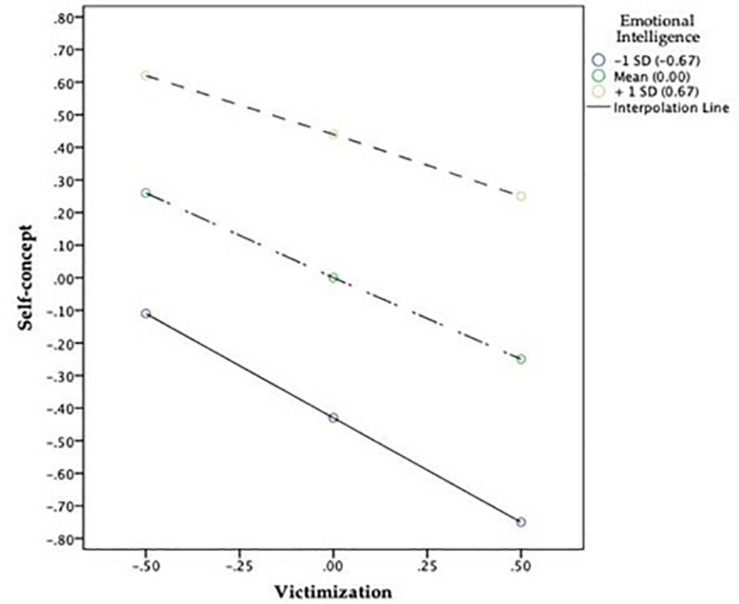
Influence of the different EI levels on the relationship between victimization and self-concept. SD, standard deviation; M, mean.

The influence of EI on self-concept was then analyzed according to the level of victimization, controlling the effects of the other variables (Figure 2). It was found that adolescents with low levels of victimization (*M* = −0.50 in the previously mean-centered variable) had a different starting point in self-concept depending on their EI level. That is, with low EI levels—one standard deviation below the mean (−0.67)—, self-concept was below the mean (−0.11) indicated for that variable (value 0), and at high levels—one standard deviation above the mean (0.67)—, self-concept was well above the mean (0.62).

This is related to the fact that EI showed a positive relationship with self-concept. Considering the interaction between EI and victimization, simple slope tests showed that for students with low EI, the effect of victimization was significant (β_simple_ = −0.65, *p* < 0.001). The effect was also significant on the medium EI group (β_simple_ = −0.501, *p* < 0.001), and regarding the high EI group (β_simple_ = −0.37, *p* < 0.001). Figure 2 indicates that the effects of victimization on self-concept differ depending on the three above-mentioned starting points, as the slopes of the three lines are different.

## Discussion

Given the importance attributed by the literature to victimization and EI regarding emotional adjustment, the present study went one step further by examining several factors that may be relevant to these relationships. Thus, the objectives of this research were, first, to examine the effect of self-concept on satisfaction with life and victimization using a mediation model. And, secondly, to analyze the role of EI in the relationship between victimization and self-concept and satisfaction with life through a moderate mediation model. The results indicated that self-concept mediated the relationship between victimization and life satisfaction, and EI moderated the negative influence of victimization on self-concept. The findings also allow to conclude that EI may moderate the relationship between victimization and life satisfaction through self-concept.

Furthermore, considering that previous research infers that emotional competence has shown to have a protective role on victims’ mental health (Quintana-Orts et al., 2019), this work has achieved to deepen into the relationship between victimization and EI on self-concept and life satisfaction. In this sense, the results indicated that both self-concept and life satisfaction were negatively associated with victimization and positively associated with EI. In line with these findings, previous studies have identified that difficulties in peer relationships, like victimization, may have a myriad of negative consequences impacting healthy development of self-concept and life satisfaction (Blakely-McClure and Ostrov, 2016; Estévez et al., 2019a; Varela et al., 2019; Cañas et al., 2020). On another hand, several studies have indicated that high EI impacts the process necessary to achieve a positive self-concept (Furqani, 2020). Some authors have tried to explain this relationship by exposing that adolescents with high EI show better emotion management, which can contribute to their perceiving themselves more positively (Fernández-Berrocal and Extremera, 2006; Martínez-Monteagudo et al., 2019). Other works have also observed an association between EI and life satisfaction, indicating that EI can act as a predictor of life satisfaction (Kong et al., 2019). However, these works do not attribute this association to better emotion management, typical of EI, but instead, they suggest that several variables may be mediating this relationship.

The first hypothesis proposed in this study postulated that self-concept would mediate the association between victimization and life satisfaction. Taking into account that victimization was directly related to life satisfaction, the results obtained confirm this hypothesis since this relationship was mediated by self-concept. These data coincide with previous studies indicating that adolescents’ assessment of their self-concept is closely related to the assessment of their lives, such that a positive self-concept is associated with high rates of life-satisfaction (Rodríguez-Fernández et al., 2016; Povedano-Diaz et al., 2020). Other works have even suggested that self-concept could predict youths’ life satisfaction (Chui and Wong, 2016; Ortuño-Sierra et al., 2019; Povedano-Diaz et al., 2020). However, these works have not focused on the context of peer victimization.

Despite the difficulty of finding studies that include analysis of emotional variables in the context of peer victimization, some research could support the results of this study, indicating that self-concept may play an indirect role in the psychological well-being of adolescent victims of bullying (Norrington, 2020). In line with this research, it is tentative to assume that self-concept might mediate the relationship between victimization and life satisfaction. However, the evidence highlights that there are also other variables, such as hope and school connectedness, that may mitigate the effect of victimization on satisfaction with life in youths (Liu et al., 2020). Despite the fact that the self-concept is not the only mediator of this relationship, there are still important gaps in the literature about that. In this regard, the current study expanded the available knowledge on the subject by including self-concept as a mediator of the relationships between victimization and satisfaction with the life of students. That is why this first mediation analysis was especially useful, by allowing to obtain pioneering results in this field.

The second hypothesis of this study proposed that EI would moderate the negative relationship of victimization with self-concept and life satisfaction. The results supported partially this hypothesis since EI only moderated the impact of victimization on self-concept. These findings are consistent with previous works, which despite being focused on cybervictimization, have analyzed the influence of EI on emotional adjustment variables, such as self-concept in victims (Estévez et al., 2020). Although these studies do not confirm the moderating effect of EI between victimization and self-concept, the literature on cyberbullying suggests that victimization could have less impact on self-perception and self-assessment when victims have high levels of EI (Extremera et al., 2018). Based on these findings and taking into account that self-concept can act as a mediator in the relationship between victimization and satisfaction with life, the moderating effect of IE could be observed indirectly on satisfaction with life, by exerting its influence on through self-concept. The literature supports the idea that higher levels of EI promote the use of adaptive strategies in uncomfortable or difficult situations, preserving the positive assessment of life in general (Lopez-Zafra et al., 2019). Therefore, it is not strange to find studies suggesting that EI may act as a buffer between maltreatment experiences and life satisfaction (Harasemiw et al., 2019) by alleviating the emotional discomfort associated with these experiences (Zhao et al., 2019).

The results obtained in this research highlight, on the one hand, the role that self-concept plays in the relationship between victimization and life satisfaction. And on the other, the importance of the effect that EI has on self-concept and satisfaction with life in the context of peer victimization. Therefore, the data of this study underline the protective and moderating factor of EI on the negative impact that victimization has on the emotional adjustment of victims.

### Strengths, Limitations, and Future Directions

To date, no such study analyzing the role of EI on the relationship of victimization with self-concept and life satisfaction has been carried out. The results of this study provide valuable insights into the moderating effect that EI has on the impact of victimization. Moreover, this study also highlights the role of self-concept as an important mechanism linking victimization and life satisfaction.

Despite the strengths of this study, it also has several limitations that must be taken into account for future research. A first limitation is based on the cross-sectional nature of the data, making it impossible to establish causal relationships between the variables examined. Future studies should carry out a longitudinal study, using measurements at different times to provide more information on causal relationships among the study variables. Second, it should be considered that, although the questionnaires were administered anonymously, self-administered instruments in adolescence could generate response bias, which affects the validity and generalizability of the data. Finally, it should be noted that the results of this study are limited to the adolescent stage from 11 to 18 years. It would be interesting to consider other samples to generalize the results to other ages or educational levels (early childhood education, primary, and higher education), or even school settings belonging to other cultures.

## Conclusion

The present study has provided empirical evidence of the negative impact of victimization on victims’ emotional adjustment. Besides, this study has also deepened our knowledge of the role of EI in the context of peer victimization, suggesting that EI should be considered as a personal resource whose effect is relevant to moderate the negative impact of victimization on emotional adjustment. Thus, this work contributes significantly to the scientific literature on peer victimization and its emotional impact in adolescence.

## Practical Implications

This article offers several implications. First, self-concept development should be an integral part of bullying programs at school, as this could become decisive for the well-being of the victims. In this line, EI should be also considered as a personal resource that is relevant to the negative consequences associated with victimization. Besides, given the important impact on emotional adjustment (i.e., negative self-concept and low life satisfaction) related to victimization, greater development of EI in victims, and youth, in general, could reduce the negative outcomes of victimization. Specifically, schools could pay more attention to the emotional development of students and promote healthy relationships among schoolmates. Moreover, it is important and necessary for students to learn about the problems associated with bullying. In consequence, schools could be used to develop school-based, integrated bullying prevention programs aimed at increasing the emotional abilities of adolescents to protect them against, or at least mitigate, the negative consequences of being a victim of bullying (Estévez et al., 2019b). Also, educational programs on the Internet and social networking sites should implement good practices so that adolescents will develop a healthy use of these communication tools to detect violence and peer victimization problems (Martínez-Ferrer et al., 2018).

## Data Availability Statement

The raw data supporting the conclusions of this article will be made available by the authors, without undue reservation.

## Ethics Statement

The studies involving human participants were reviewed and approved by the Ethics Committee of the Miguel Hernández University, besides complying with the ethical values required for research with human beings and respecting the basic principles included in the Helsinki Declaration. Written informed consent to participate in this study was provided by the participants’ legal guardian/next of kin.

## Author Contributions

All authors contributed to the development of this study and provided quality checks in data analyses and the writing of the final manuscript. JE and DA were responsible for data analyses and interpretation. EE obtained funding and was also responsible for data collection and study supervision. EC was responsible for data collection and the first draft of the manuscript.

## Conflict of Interest

The authors declare that the research was conducted in the absence of any commercial or financial relationships that could be construed as a potential conflict of interest.

## References

[B1] Atienza F. L. Pons D. Balaguer I. Merita M. G. (2000). Propiedades psicométricas de la Escala de satisfacción con la Vida en adolescentes [Psychometric properties of the Life Satisfaction Scale in adolescents]. *Psicothema* 12 314–319.

[B2] Blakely-McClure S. J. Ostrov J. M. (2016). Relational aggression, victimization and self-concept: testing pathways from middle childhood to adolescence. *J. Youth Adolesc.* 45 376–390. 10.1007/s10964-015-0357-2 26419234

[B3] Brislin R. W. (1986). “The wording and translation of research instruments,” in *Field Methods in Cross-Cultural Psychology*, eds Lonner W. J. Berry J. W. (Newbury Park, CA: Sage Publications), 137–164.

[B4] Callaghan M. Kelly C. Molcho M. (2015). Exploring traditional and cyberbullying among Irish adolescents. *Int. J. Public Health* 60 199–206. 10.1007/s00038-014-0638-7 25540816

[B5] Callaghan M. Kelly C. Molcho M. (2019). Bullying and bystander behaviour and health outcomes among adolescents in Ireland. *J, Epidemiol. Commun. Health* 73 416–421. 10.1136/jech-2018-211350 30765490

[B6] Cañas E. Estévez E. León-Moreno C. Musitu G. (2020). Loneliness, family communication, and school adjustment in a sample of cybervictimized adolescents. *Int. J. Public Health* 17 335–348. 10.3390/ijerph17010335 31947793 PMC6982055

[B7] Cañas E. Estévez E. Martínez-Monteagudo M. Delgado B. (2019). Emotional adjustment in victims and perpetrators of cyberbullying and traditional bullying. *Soc. Psychol. Educ.* 23 917–942. 10.1007/s11218-020-09565-z

[B8] Carrascosa L. Cava M. J. Buelga S. (2018). Perfil psicosocial de adolescentes españoles agresores y víctimas de violencia de pareja [Psychosocial profile of spanish adolescent aggressors and victims of dating violence]. *Univ. Psychol.* 17 1–10. 10.11144/Javeriana.upsy17-3.ppae

[B9] Chui W. H. Wong M. Y. (2016). Gender differences in happiness and life satisfaction among adolescents in Hong Kong: relationships and self-concept. *Soc. Indic. Res.* 125 1035–1051. 10.1007/s11205-015-0867-z

[B10] De la Barrera U. Schoeps K. Gil-Gómez J. A. Montoya-Castilla I. (2019). Predicting adolescent adjustment and well-being: the interplay between socio-emotional and personal factors. *Int. J. Env. Res. Public Health* 16:4650. 10.3390/ijerph16234650 31766641 PMC6926821

[B11] Diener E. Suh E. M. Lucas R. E. Smith H. L. (1999). Subjective well-being: three decades of progress. *Psychol. Bull.* 125 276–302. 10.1007/s11205-015-0867-z

[B12] Diener E. D. Emmons R. A. Larsen R. J. Griffin S. (1985). The satisfaction with life scale. *J. Pers. Assess.* 49 71–75. 10.1207/s15327752jpa4901_13 16367493

[B13] Domínguez E. Martín P. Martín-Albo J. Núñez J. L. León J. (2010). Translation and validation of the Spanish version of the “Échelle de satisfaction des besoins psychologiques” in the sports context. *Span. J. Psychol.* 13 1010–1020. 10.1017/s1138741600002651 20977048

[B14] Domínguez-García E. Fernández-Berrocal P. (2018). The association between emotional intelligence and suicidal behavior: a systematic review. *Front. Psychol.* 9:2380. 10.3389/fpsyg.2018.02380 30555393 PMC6284019

[B15] Estévez E. Estévez J. F. Segura L. Suárez C. (2019a). The influence of bullying and cyberbullying in the psychological adjustment of victims and aggressors in adolescence. *Int. J. Env. Res. Public Health* 16:2080. 10.3390/ijerph16122080 31212830 PMC6616482

[B16] Estévez E. Flores E. Estévez J. F. Huéscar E. (2019b). Programas de intervención en acoso escolar y ciberacoso en educación secundaria con eficacia evaluada: una revisión sistemática. *Rev. Lat. Am. Psicol.* 51 210–225. 10.14349/rlp.2019.v51.n3.8

[B17] Estévez E. Jiménez T. Segura L. (2019c). Emotional intelligence and empathy in aggressors and victims of school violence. *J. Educ. Psychol.* 111 488–496. 10.1037/edu0000292

[B18] Estévez J. F. Cañas E. Estévez E. (2020). The impact of cybervictimization on psychological adjustment in adolescence: analyzing the role of emotional intelligence. *Int. J. Environ. Res. Public Health* 17:3693. 10.3390/ijerph17103693 32456261 PMC7277426

[B19] Extremera N. Quintana-Orts C. Mérida-López S. Rey L. (2018). Cyberbullying victimization, self-esteem and suicidal ideation in adolescence: does emotional intelligence play a buffering role? *Front. Psychol.* 9:367. 10.3389/fpsyg.2018.00367 29623058 PMC5874900

[B20] Fairchild A. J. Mcquillin S. D. (2010). Evaluating mediation and moderation effects in school psychology: a presentation of methods and review of current practice HHS public access. *J. Sch. Psychol.* 48 53–84. 10.1016/j.jsp.2009.09.001 20006988 PMC5488867

[B21] Fernández-Berrocal P. Extremera N. (2006). Emotional intelligence: a theoretical and empirical review of its first 15 years of history. *Psicothema.* 18 7–12. 17295952

[B22] Frazier P. A. Tix A. P. Barron K. E. (2004). Testing moderator and mediator effects in counseling psychology research. *J. Counsel. Psychol.* 51 115–134. 10.1037/0022-0167.51.1.115

[B23] Furqani N. N. (2020). “The role of emotional intelligence in adolescent development,” in *Advances in Social Science, Education and Humanities Research*, eds Striełkowski W. Cheng J. (Paris: Atlantis Press), 277–280.

[B24] García F. Musitu G. (1999). *Autoconcepto Forma 5.* Madrid: Tea.

[B25] García F. J. Carrero V. E. Marande G. Musitu G. (2017). Understanding rejection between first-and-second-grade elementary students through reasons expressed by rejecters. *Front. Psychol.* 8:462. 10.3389/fpsyg.2017.00462 28421008 PMC5378718

[B26] García L. Quintana-Orts C. Rey L. (2020). Cibervictimización y satisfacción vital en adolescentes: la inteligencia emocional como variable mediadora [Cybervictimization and life satisfaction in adolescents: the emotional intelligence as a mediating variable]. *Rev. Psicol. Clín. Niños Adolesc.* 7 2020–2058. 10.21134/rpcna.2020.07.1.5

[B27] Gini G. Marino C. Pozzoli T. Holt M. (2018). Associations between peer victimization, perceived teacher unfairness, and adolescents’ adjustment and well-being. *J. Sch. Psychol.* 67 56–68. 10.1016/j.jsp.2017.09.005 29571535

[B28] Goleman D. (1995). *Emotional Intelligence.* New York, NY: Bantam.

[B29] Gómez-Baya D. Mendoza R. Paino S. Gaspar M. (2017). Perceived emotional intelligence as a predictor of depressive symptoms during mid-adolescence: a two-year longitudinal study on gender differences. *Pers. Indiv. Differ.* 104 303–312. 10.1016/j.paid.2016.08.022

[B30] Hancock G. R. Stapleton L. M. Muller R. O. (2018). “The reviewer’s guide to quantitative methods in the social sciences,” in *The Reviewer’s Guide to Quantitative Methods in the Social Sciences*, 2nd Edn, eds Hancock G. R. Stapleton L. M. Muller R. O. (New York, NY: Routledge), 10.4324/9781315755649

[B31] Harasemiw O. Newall N. Mackenzie C. S. Shooshtari S. Menec V. (2019). Is the association between social network types, depressive symptoms and life satisfaction mediated by the perceived availability of social support? A cross-sectional analysis using the canadian longitudinal study on aging. *Aging Ment. Health* 23 1413–1422. 10.1080/13607863.2018.1495176 30406668

[B32] Hayes A. F. (2018). *Introduction to Mediation, Moderation, and Conditional Process Analysis*, 2nd Edn, New York, NY: Guilford Press.

[B33] Kardefelt-Winther D. Maternowska C. (2020). Addressing violence against children online and offline. *Nat. Hum. Behav.* 4 227–230. 10.1038/s41562-019-0791-3 31831869

[B34] Kong F. Gong X. Sajjad S. Yang K. Zhao J. (2019). How is emotional intelligence linked to life satisfaction? The mediating role of social support, positive affect and negative affect. *J. Happ. Stud.* 20 2733–2745. 10.1007/s10902-018-00069-4

[B35] Låftman S. B. Modin B. (2017). Peer victimization among classmates—associations with students’ internalizing problems, self-esteem, and life satisfaction. *Int. J. Environ. Res. Public Health* 14:1218. 10.3390/ijerph14101218 29027932 PMC5664719

[B36] Lázaro-Visa S. Palomera R. Briones E. Fernández-Fuertes A. Fernández-Rouco N. (2019). Bullied adolescent’s life satisfaction: personal competencies and school climate as protective factors. *Front. Psychol.* 10:1691. 10.3389/fpsyg.2019.01691 31379695 PMC6657649

[B37] Liu Y. Carney J. V. Kim H. Hazler R. J. Guo X. (2020). Victimization and students’ psychological well-being: the mediating roles of hope and school connectedness. *Child. Youth Serv. Rev.* 108:104674 10.1016/j.childyouth.2019.104674

[B38] Lomas J. Stough C. Hansen K. Downey L. A. (2012). Brief report: emotional intelligence, victimisation and bullying in adolescents. *J. Adolesc.* 35 207–211. 10.1016/j.adolescence.2011.03.002 21470670

[B39] Lopez-Zafra E. Ramos-Álvarez M. M. El Ghoudani K. Luque-Reca O. Augusto-Landa J. M. Zarhbouch B. (2019). Social support and emotional intelligence as protective resources for well-being in moroccan adolescents. *Front. Psychol.* 10:1529. 10.3389/fpsyg.2019.01529 31354568 PMC6635474

[B40] Malhi P. Bharti B. Sidhu M. (2014). Aggression in schools: psychosocial outcomes of bullying among Indian adolescents. *Indian J. Pediatr.* 81 1171–1176. 10.1007/s12098-014-1378-7 24659440

[B41] Martínez-Ferrer B. Moreno D. Musitu G. (2018). Are adolescents engaged in the problematic use of social networking sites more involved in peer aggression and victimization? *Front. Psychol.* 9:801. 10.3389/fpsyg.2018.00801 29896139 PMC5987195

[B42] Martínez-Monteagudo M. C. Inglés C. J. Suriá R. Lagos N. Delgado B. García-Fernández J. M. (2019). Emotional intelligence profiles and self-concept in Chilean adolescents. *Curr. Psychol.* 1–8. 10.1007/s12144-019-00350-6

[B43] Mehari K. R. Farrell A. D. (2015). The relation between peer victimization and adolescents’ well-being: the moderating role of ethnicity within context. *J. Res. Adolesc.* 25 118–134. 10.1111/jora.12095

[B44] Méndez-Giménez A. Cecchini-Estrada J. A. Fernández-Río J. Mendez-Alonso D. Prieto-Saborit J. A. (2017). Metas de logro 3×2, motivación autodeterminada y satisfacción con la vida en educación secundaria [Achievement goals 3×2, self-determined motivation and life satisfaction in secondary education]. *Rev. Psicodidact.* 22 150–156. 10.1016/j.psicod.2017.05.001

[B45] Menesini E. Salmivalli C. (2017). Bullying in schools: the state of knowledge and effective interventions. *Psychol. Health Med.* 22 240–253. 10.1080/13548506.2017.1279740 28114811

[B46] Miranda R. Oriol X. Amutio A. Ortúzar H. (2019). Adolescent bullying victimization and life satisfaction: Can family and school adult support figures mitigate this effect?. *Revista de Psicodidáctica.* 24 39–45. 10.1016/j.psicoe.2018.07.001

[B47] Mynard H. Joseph S. (2000). Development of the multidimensional peer-victimization scale. *Aggress. Behav.* 26 169–178. 10.1002/(sici)1098-2337(2000)26:2<169::aid-ab3>3.0.co;2-a

[B48] Norrington J. (2020). Adolescent peer victimization, self-concept, and psychological distress in emerging adulthood. *Youth Soc.* 1:1–23. 10.1177/0044118x20910938

[B49] Navarro R. Yubero S. Larrañaga E. (2015). Psychosocial risk factors for involvement in bullying behaviors: Empirical comparison between cyberbullying and social bullying victims and bullies. *Sch. Ment. Health*, 7, 235–248. 10.1007/s12310-015-9157-9

[B50] Ortuño-Sierra J. Aritio-Solana R. Chocarro de Luis E. Nalda F. N. Fonseca-Pedrero E. (2019). Subjective well-being in adolescence: new psychometric evidences on the satisfaction with life scale. *Eur. J. Dev. Psychol.* 16 236–244. 10.4324/9781351231879-1

[B51] Povedano-Diaz A. Muñiz-Rivas M. Vera-Perea M. (2020). Adolescents’ life satisfaction: the role of classroom, family, self-concept and gender. *Int. J. Environ. Res. Public Health* 17 19–31. 10.3390/ijerph17010019 31861387 PMC6982278

[B52] Quintana-Orts C. Rey L. Mérida-López S. Extremera N. (2019). What bridges the gap between emotional intelligence and suicide risk in victims of bullying? A moderated mediation study. *J. Affect. Disord.* 245 798–805. 10.1016/j.jad.2018.11.030 30699862

[B53] Ramos-Díaz E. Rodríguez-Fernández A. Axpe I. Ferrara M. (2019). perceived emotional intelligence and life satisfaction among adolescent students: the mediating role of resilience. *J. Happ. Stud.* 20 2489–2506. 10.1007/s10902-018-0058-0

[B54] Rey L. Mérida-López S. Sánchez-Álvarez N. Extremera N. (2019). When and how do emotional intelligence and flourishing protect against suicide risk in adolescent bullying victims? *Int. J. Environ. Res. Public Health* 16 2114–2128. 10.3390/ijerph16122114 31207915 PMC6616872

[B55] Rodríguez-Fernández A. Ramos-Díaz E. Ros I. Fernández-Zabala A. Revuelta L. (2016). Subjective well-being in adolescence: the role of resilience, self-concept and perceived social support. *Suma Psicol.* 23 60–69.

[B56] Salovey P. Mayer J. D. (1990). Emotional intelligence. *Imagin. Cogn. Pers.* 9 185–211. 10.1142/9789811203558_0009

[B57] Salovey P. Mayer J. D. Goldman S. L. Turvey C. Palfai T. P. (1995). “Emotional attention, clarity, and repair: exploring emotional intelligence using the trait meta-mood scale,” in *Emotion, Disclosure and Health*, ed. Pennebaker J. W. (Washington, DC: American Psychological Association), 125–154. 10.1037/10182-006

[B58] Sun P. Wang S. Kong F. (2014). Core self-evaluations as mediator and moderator of the relationship between emotional intelligence and life satisfaction. *Soc. Indic. Res.* 118 173–180. 10.1007/s11205-013-0413-9

[B59] Suriá-Martínez R. Ortigosa J. M. Riquelme A. (2019). Emotional intelligence profiles of university students with motor disabilities: differential analysis of self-concept dimensions. *Int. J. Environ. Res. Public Health* 16 4073–4084. 10.3390/ijerph16214073 31652742 PMC6862470

[B60] Turner H. A. Shattuck A. Finkelhor D. Hamby S. (2017). Effects of poly-victimization on adolescent social support, self-concept, and psychological distress. *J. Interpers. Viol.* 32 755–780. 10.1177/0886260515586376 26033616

[B61] UNESCO (2018). *School Violence and Bullying: Global Status and Trends, Drivers and Consequences.* Paris: United Nations Educational Scientific and Cultural Organization.

[B62] Varela J. J. Guzmán J. Alfaro J. Reyes F. (2019). Bullying, cyberbullying, student life satisfaction and the community of chilean adolescents. *Appl. Res. Qual. Life* 14 705–720. 10.1007/s11482-018-9610-7

[B63] Varela J. J. Zimmerman M. A. Ryan A. M. Stoddard S. A. Heinze J. E. Alfaro J. (2017). Life satisfaction, school satisfaction, and school violence: a mediation analysis for Chilean adolescent victims and perpetrators. *Child Indic. Res.* 11 487–505. 10.1007/s12187-016-9442-7

[B64] Weng X. Chui W. H. Liu L. (2017). Bullying behaviors among macanese adolescents—association with psychosocial variables. *Int. J. Environ. Res. Public Health* 14 887–899. 10.3390/ijerph14080887 28783110 PMC5580591

[B65] Wu L. Zhang D. Su Z. Hu T. (2015). Peer victimization among children and adolescents: a meta-analytic review of links to emotional maladjustment. *Clin. Pediatr.* 54 941–955. 10.1177/0009922814567873 25644646

[B66] Zhao J. Peng X. Chao X. Xiang Y. (2019). Childhood maltreatment influences mental symptoms: the mediating roles of emotional intelligence and social support. *Front. Psychiatry* 10:415. 10.3389/fpsyt.2019.00415 31316399 PMC6611427

[B67] Zych I. Farrington D. P. Llorent V. J. Ttofi M. M. (2017). *Protecting Children Against Bullying and its Consequences.* Berlin: Springer.

